# Understanding SUMO-mediated adaptive responses in plants to improve crop productivity

**DOI:** 10.1042/EBC20210068

**Published:** 2022-08-05

**Authors:** Lisa Clark, Kawinnat Sue-Ob, Vaishnavi Mukkawar, Andrew R. Jones, Ari Sadanandom

**Affiliations:** 1 Department of Biosciences, Durham University, Stockton Rd, Durham DH1 3LE, U.K.; 2 Department of Biochemistry and Systems Biology, Institute of System, Molecular and Integrative Biology, University of Liverpool, Liverpool L69 7BE, U.K.

**Keywords:** abiotic, biotic, plants, protein modification, stress

## Abstract

The response to abiotic and biotic stresses in plants and crops is considered a multifaceted process. Due to their sessile nature, plants have evolved unique mechanisms to ensure that developmental plasticity remains during their life cycle. Among these mechanisms, post-translational modifications (PTMs) are crucial components of adaptive responses in plants and transduce environmental stimuli into cellular signalling through the modulation of proteins. SUMOylation is an emerging PTM that has received recent attention due to its dynamic role in protein modification and has quickly been considered a significant component of adaptive mechanisms in plants during stress with great potential for agricultural improvement programs. In the present review, we outline the concept that small ubiquitin-like modifier (SUMO)-mediated response in plants and crops to abiotic and biotic stresses is a multifaceted process with each component of the SUMO cycle facilitating tolerance to several different environmental stresses. We also highlight the clear increase in SUMO genes in crops when compared with *Arabidopsis thaliana.* The SUMO system is understudied in crops, given the importance of SUMO for stress responses, and for some SUMO genes, the apparent expansion provides new avenues to discover SUMO-conjugated targets that could regulate beneficial agronomical traits.

## Molecular mechanisms underpinning adaptive responses in plants

Global warming and climate change have become one of the most challenging environmental issues to face humankind. Significant changes in temperature and weather patterns have led to an increase in the occurrence of extreme climate events and the destruction of a vulnerable agricultural system [1]. As plants are sessile organisms restricted to their site of germination, an effective adaptive response is essential to ensure their survival in an ever-changing diverse environment; therefore, plants have evolved unique mechanisms to ensure that developmental plasticity remains during their life cycle [2,3]. Among these mechanisms, post-translational modifications (PTMs) constitute crucial master regulators of adaptive signalling pathways in plants and transduce environmental stimuli into cellular signalling through the modulation of protein function [4].

There are more than 400 different types of PTMs involved in the modulation of protein function. According to the dbPTM database, phosphorylation, acetylation, and ubiquitination are considered the most common and represent more than 90% of all reported PTM sites [5]. Of these, ubiquitination is the most important PTM due to its reversibility, versatility to take effect on all 20 amino acids and its critical role in the intracellular degradation of proteins via the ubiquitin-proteasome pathway [6]. In *Arabidopsis thaliana (A. thaliana)*, the ubiquitin-proteasome system (UPS) encodes for two ubiquitin E1-activating enzymes, up to 45 E2-conjugating enzymes and an estimated 1400 E3 ligases [7,8]. The UPS is well-documented in the literature and is shown to play an integral role in facilitating cellular changes required in plant adaptation to environmental abiotic and biotic stresses [9,10]. Likened to ubiquitination, SUMOylation is an emerging PTM that has received widespread attention due to its dynamic role in protein modification and has quickly been considered significant in understanding the molecular adaptive mechanisms in plants during stress survival and its potential utilization in the agriculture [11–14].

## Small ubiquitin-like modifier cycle

As the majority of information regarding SUMO is mostly known in the model organism *A. thaliana*, we will describe the role of SUMO in this species for clarity. SUMOylation is an essential PTM involving members of the small ubiquitin-like modifier (SUMO) protein family [15]. In plants, SUMO isoforms are 11-kDa proteins and are involved in several essential processes of plant development [16,17]. In the study of the SUMO system, the model organism, *A. thaliana*, is the most advanced plant due to its genetically tractable nature [18]. *A. thaliana* encodes for eight SUMO isoforms; however, only *AtSUMO1*, *AtSUMO2*, *AtSUMO3*, and *AtSUMO5* are expressed—with *AtSUMO1* and *AtSUMO2* expression levels highest [14,19,20]. *AtSUMO1* and *AtSUMO2* genes share an 89% sequence similarity, whereas *AtSUMO3* and *AtSUMO5* are more distantly related to *AtSUMO1* with a sequence similarity of 48 and 35%, respectively [21]. Not only are the SUMO isoforms of *A. thaliana* distantly related, but they have also acquired their own individual patterns of expression and functional diversification [22].

*AtSUMO1* and *AtSUMO2* exhibit similar functions in *A. thaliana* and are considered better conjugation substrates than *AtSUMO3* [23]. Similarly, it is also documented in the literature that the known SUMO ULP proteases in *A. thaliana* exhibit higher (iso)peptidase activity to conjugates of *AtSUMO1/2* compared with *AtSUMO3* [21]. A clear difference between the role of these isoforms is also evident, where Kurepa et al. [24] observed that SUMO conjugation levels of *AtSUMO1/2* were increased compared with *AtSUMO3* in *A. thaliana* seedlings exposed to a variety of different abiotic stress conditions. In contrast, unlike *AtSUMO1/2*, *AtSUMO3* conjugates are increased by salicylic acid (SA) and when overexpressed enhance resistance to Pst DC3000 [23]. Similarly, *AtSUMO3* has also been shown to mediate the SUMOylation of the master transcription regulator of SA-signaling *nonexpressor of pathogenesis related 1* (*NPR1)*, demonstrating that *AtSUMO3* is a positive regulator of immunity [25]. In the case of *AtSUMO5*, its function remains unclear; however, initial results suggest that it is more divergent than *AtSUMO1/2* and has potentially neofunctionalized [21].

SUMOylation occurs in a manner similar to ubiquitination through a biochemical cascade catalyzed by a set of well-conserved enzymes—conjugating to a lysine residue in the target protein. Unlike ubiquitination, the process of SUMOylation comprises a smaller group of proteins. In *A. thaliana*, there is one E1 SUMO-activating enzyme—(*AtSAE1/2*), one E2 SUMO-conjugating enzyme—(*AtSCE1*), two E3 ligases—*SAP and MIZ1* (*AtSIZ1*) and *high ploidy2* (*AtMMS21/HPY2*), and two E4 ligases—*protein inhibitor of activated state like1* and *2* (*AtPIAL1* and *AtPIAL2*)—Table 1. In contrast with ubiquitin, SUMOylation can have several effects on target proteins. These include protecting lysine residues prone to ubiquitination, preventing protein degradation, changing the localization of target proteins, and modifying the interaction between proteins in several cellular processes to alleviate stress-induced damage [4,26].

**Table 1:** SUMO machinery summary

Species	SUMO	E1	E2	E3	E4	Desi	ULP
*A. thaliana*	AtSUMO1 (At4g26840)	AtSAE1a (At5g55856)	AtSCE1 (At3g57870)	AtSIZ1 (At5g60410)	AtPIAL1 (At1g08910)	AtDESI1 (At3g07090)	AtOTS1 (At1g60220)
	AtSUMO2 (At5g55160)	AtSAE1b (At5g50580/ Atg50680)		AtHPY2 (At3g15150)	AtPIAL2 (At5g41580)	AtDESI2a (At4g25660)	AtOTS2 (At1g10570)
	AtSUMO3 (At5g55170)	AtSAE2 (At2g21470)				AtDESI2b (At4g25680)	AtESD4 (At4g15880)
	AtSUMO4 (At5g48700)					AtDESI3a (At1g47740)	AtELS1 (At3g06910)
	AtSUMO5 (At2g32765)					AtDESI3b (At2g25190)	AtELS2 (At4g00690)
	AtSUMO6 (At5g48710)					AtDESI3c (At5g25170)	AtFUG1 (At3g48480)
	AtSUMO7 (At5g55855)					AtDESI4a (At4g17486)	AtSPF1 (At1g09730)
	AtSUMO8 (At5g55856)					AtDESI4b (At5g47310)	AtSPF2 (At4g33620)
*O. sativa*	OsSUMO1 (Os01g68950)	OsSAE1 (Os11g30410)	OsSCE1 (Os10g39120)	OsSIZ1 (Os05g03430)	-	-	OsOTS1 (Os06g29310)
	OsSUMO2 (Os01g68940)	OsSAE2 (Os07g39780)	OsSCE2 (Os04g49130)	OsSIZ2 (Os03g50980)			OsOTS2 (Os12g41380)
	OsSUMO3 (Os07g38690)		OsSCE3 (Os03g03130)	OsMMS21/HYP2 (Os05g48880)			OsOTS3 (Os01g53630)
	OsSUMO4 (OS07G38660)						OsELS2/OsESD4a (Os03g29630)
	OsSUMO5 (Os07g38650)						OsFUG1/OsESD4b (Os03g22400)
							OsESD4c (Os01g25370)
	OsSUMO6 (Os07g0574300, LOC_Os07g38660)						OsSPF1 (Os05g11770)
							OsPROa (Os11g10780)
*Z. mays*	ZmSUMO1a (GRMZM2G053898)	ZmSAE1 (GRMZM2G149108)	ZmSCE1a (GRMZM2G063931 _T01)	ZmSIZ1a (GRMZM2G155123)	ZmPIAL1 (GRMZM2G075582)	-	ZmESD4a (GRMZM2G088653)
	ZmSUMO1b (GRMZM2G082390)	ZmSAE2a (GRMZM2G129575)	ZmSCE1b (GRMZM2G070047 _T01)	ZmSIZ1b (GRMZM2G155123)			ZmESD4b (GRMZM2G012601)
	ZmSUMO2 (GRMZM2G305196)	ZmSAE2b (Scaffold 252)	ZmSCE1c (GRMZM2G312693 _T01)	ZmSIZ1c (GRMZM2G173770/ GRMZM2G002999)			ZmESD4c (GRMZM5G849959)
			ZmSCE1d (GRMZM2G163398 _T01)	ZmMMS21 (GRMZM2G022065)			ZmESD4d (GRMZM2G010505)
			ZmSCE1e (GRMZM2G038851 _T01)				ZmOTS1a (GRMZM2G072939)
			ZmSCE1f (GRMZM2G341089 _T02)				ZmOTS1b (GRMZM2G174667)
			ZmSCE1g (GRMZM2G433968 _T01)				ZmOTS1c (GRMZM5G5886883/ GRMZM2G1425533)
							ZmOTS1d (GRMZM2G351786)
							ZmOTS1e (GRMZM2G432931)
							ZmB2 (GRMZM2G177324)
							ZmPROa (GRMZM2G545326)
*S. lycopersicum*	SiSUMO1 (Solyc07g064880)	SiSAE1a (Solyc03g019730)	SiSCEa (Solyc03g044260)	SiSIZ1a (Solyc11g069160)	SiPIAL1 (Solyc08g008130)	-	SiPROa (Solyc00g007260)
	SiSUMO2 (Solyc12g006010)	SiSAE1b (Solyc06g072080)	SiSCEb (Solyc02g093110)	SiSZ1b (Solyc06g010000)			SiPROb (Solyc11g072220)
	SiSUMO3 (Solyc07g049360)	SiSAE2 (Solyc01g109960)	SiSCEc (Solyc12g088680)	SiMMS21 (Solyc07g062780)			SiOTSa (Solyc04g026200)
	SiSUMO4 (Solyc09g059970)		SiSCEd (Solyc04g078620)				SiOTSb (Solyc05g005630)
	SiSUMO5 (Solyc09g091890)		SiSCEe (Solyc03g112720)				SiB2a (Solyc01g105830)
			SiSCEf (Solyc07g021660)				SiB2b (Solyc11g017040)
							SiESD4a (Solyc01g066830)
							SiESD5a (Solyc12g099530)
*S. tuberosum*	StSUMO1 (PGSC0003DMG 400023103)	-	StSCE1a (PGSC0003DMG 400007950)	-	-	-	-
	StSUMO2 (PGSC0003DMG 400022207)		StSCE2 (PGSC0003DMG 400009844)				
	StSUMO3 (PGSC0003DMG 400000665)		StSCE3 (PGSC0003DMG 400015405)				
	StSUMO4 (PGSC0003DMG 4000138990		StSCE4 (PGSC0003DMG 400006481)				
	StSUMO5 (PGSC0003DMG 400031298)		StSCE5 (PGSC0003DMG 400018161)				
	StSUMO6 (PGSC0003DMG 400030378)		StSCE6 (PGSC0003DMG 400033039)				
	StSUMO7 (PGSC0003DMG 400031297)		StSCE7 (PGSC0003DMG 400018082)				
			StSCE8 (PGSC0003DMG 401006935)				
			StSCE9 (PGSC0003DMG 402018159)				
*G. max*	GmSUMO1 (Glyma08g320500)	GmSAE1a (Glyma08g011400)	GmSCEa (Glyma11g053300)	GmSIZ1a (Glyma12g071300)	GmPIAL1 (Glyma11g020900)	-	GmPROa (Glyma04g193100)
	GmSUMO2 (Glyma18g165200)	GmSAE1b (Glyma05g204000)	GmSCEb (Glyma01g188900)	GmSIZ1b (Glymau020100)	GmPIAL2 (Glyma01g222500)		GmOTSa (Glyma18g137700)
	GmSUMO3 (Glyma08g350600)	GmSAE2a (Glyma13g201500)	GmSCEc (Glyma17g169700)	GmSIZ1c (Glyma12g170900)			GmOTSb1 (Glyma08g287800)
	GmSUMO4 (Glyma08g111700)	GmSAE2b (Glyma12g236000)	GmSCEd (Glyma05g091100)	GmSIZ1d (Glyma13g328100)			GmOTSb2 (Glyma08g287700)
	GmSUMO5 (Glyma08g111800)			GmMMS21 (Glyma13g273500)			GmB2a (Glyma06g095600)
	GmSUMO6 (Glyma05g154000)						GmB2b (Glyma04g093800)
							GmB2c (Glyma15g058100)
							GmB2d (Glyma13g256800)
							GmB2e (Glyma02g214100)
							GmESD4a (Glyma15g148700)
							GmESD4b (Glyma09g044400)
							GmESD4c (Glyma17g027100)
							GmESD4d (Glyma17g246900)

Collective summary of all the SUMO components involved in the SUMO cycle of *A. thaliana*, rice (*O. sativa*), maize (*Z. mays*), tomato (*S. lycopersicum*), potato (*S. tuberosum*), and soybean (*G. max*) [12,42].

Multiple studies have demonstrated the importance of the different SUMO components in all eukaryotes from mammals and plants to single-cell yeasts over the past two decades. Deletion of the first identified SUMO homolog *suppressor of mif two3* (*SMT3*) in the budding yeast, *Saccharomyces cerevisiae*, has shown to result in a loss of cell viability [27]. Similarly, the introduction of single *Atsae1*, *Atsae2* and *Atsce1* mutants and double *Atsumo1 Atsumo2* and *Atsiz1 Atmms21* mutants in *A. thaliana* plants is embryo lethal [28]. Aside from development, the adaptive response of SUMO proteins has also shown to be beneficial during their response to stress due to their ability to be both rapid and reversible upon the introduction of stress stimuli. This is evident in *A. thaliana* where Kurepa et al. [24] observed that heat induction initiates the accumulation of *AtSUMO1/2* conjugates as early as 2 min, following a temperature shift and upon returning the plants to normal growth conditions: the increase in SUMO conjugation decreased and the pool of free SUMO increased to prestressed levels.

The process of SUMOylation in *A. thaliana* requires the attachment of SUMO proteins to a lysine residue in a target substrate using several enzymatic reactions. First, free SUMO is translated as a precursor that undergoes maturation by SUMO proteases. Currently, all SUMO proteases that have been identified in the SUMO system are cysteine proteases and are members of either the *AtULP* or *AtDeSI* gene families; however, only *AtULP* proteases have been shown to play a role in maturation [2]. During maturation, SUMO *AtULP* proteases recognize a C-terminal diglycine motif and cleave off approximately ten amino acids—exposing the diglycine motif [21]. The SUMO protein is then catalyzed into activation by the SUMO-activating enzyme (SUMO E1), which consists of a heterodimer of a regulatory subunit *AtSAE1a/AtSAE1b* and a catalytic subunit *AtSAE2* [29]. For this reaction to take place an ATP molecule is hydrolyzed, forming a high-energy thioester bond between the cysteine residue in the catalytic subunit *AtSAE2* and the exposed glycine residue in the SUMO protein [30]. The activated SUMO protein is then transferred in a transesterification reaction from *AtSAE2* to a cysteine residue in the E2 conjugation enzyme—*AtSCE1* to form a SUMO-SCE1 thioester complex [31,32]. The SUMO-SCE1 complex then catalyzes the process of SUMOylation using an isopeptide bond between the C-terminal diglycine residue of the SUMO protein and a lysine residue in the target protein [33].

**Figure 1:**
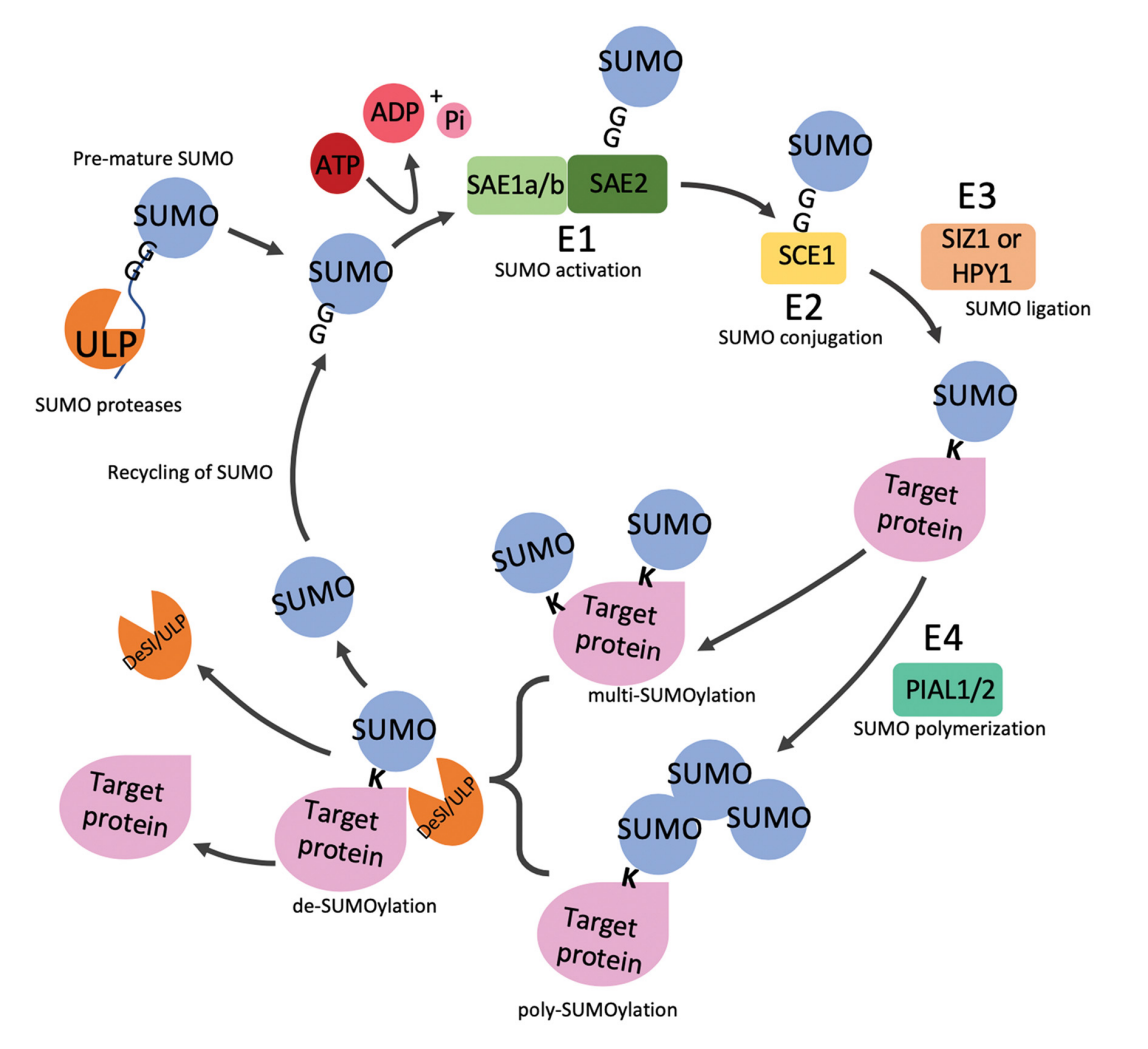
The SUMO cycle—a brief overview The SUMO cycle begins with free precursor SUMO undergoing maturation via a SUMO ULP protease—cleaving off the C-terminus exposing a diglycine motif. Mature SUMO is then activated by a hydrolyzed ATP molecule and a SUMO E1 enzyme—a heterodimer of *AtSAE1a/b* and *AtSAE2* [21]. The activated SUMO is transferred from *AtSAE2* to *AtSCE1*, an E2 conjugation enzyme—forming a SUMO-*AtSCE1* thioester complex, which catalyzes the process of SUMOylation onto a target protein [29]. SUMO E3 ligases aid in the transfer of SUMO proteins from *AtSCE1* onto the lysine residue of target proteins [25]. SUMO E4 is a further step in the SUMO cycle and promotes the formation of SUMO chains [34,35]. Finally, SUMO proteases cleave SUMO from target proteins via a process called deSUMOylation to create pools of free SUMO, therefore, making the process of SUMOylation reversible [2].

Although the process of SUMO conjugation can be directly catalyzed by the E2 SUMO-SCE1 complex, this interaction alone is often insufficient; therefore, E3 ligases are required to aid in the transfer [26]. A further step also identified involves SUMO E4 proteins, which are specialized E3 ligases that exhibit elongase activity and promote the formation of SUMO chains in an *AtSCE1*-dependent manner onto target proteins [33,35]. In the final step, SUMO proteases cleave SUMO from target proteins to create pools of free SUMO, therefore, making SUMOylation a reversible process [2]—Figure 1.

## The role of SUMO in adaptive responses against abiotic and biotic stresses in *A. thaliana* and major crops

The first implications of the role of SUMO in response to abiotic and biotic stresses were discovered by detecting an increased abundance of SUMO conjugates in protein extracts of stressed plants [28]. In plants, the SUMO-conjugate accumulation is not only influenced by the induction of a stress stimulus but also by the severity and duration of the stress and more crucially differential regulation of one or more of the components that comprise the SUMO cycle [36–39].

## E1-activating enzymes and stress responses

In *A. thaliana*, the SUMO E1-activating enzyme is made up of three subunits—*AtSAE1a*, *AtSAE1b*, and *AtSAE2*. It is suggested that the E1-activating enzyme plays one of the most essential roles in the SUMO cycle due to its location within the first step of the conjugation pathway. The majority of the E1 function is located within the catalytic *AtSAE2* subunit, which has been shown to play a critical role in early plant development where *Atsae2* mutant plants are embryo lethal [14,31]. There is limited information on the role of *AtSAE2* in response to plant stress, but this is likely due to *Atsae2* mutants been embryo lethal.

Initially, it was first speculated that *AtSAE1* may be redundant and unessential—with initial results indicating that T-DNA *Atsae1a* mutants are viable. However, in recent studies, this has since been disproven and a role for *AtSAE1* in the regulation of SUMO conjugation during abiotic stress has been proposed. Mutant *Atsae1a A. thaliana* plants, when exposed to heat and drought stresses, displayed phenotypic defects and reduced SUMO-conjugate accumulation. These results suggest that *AtSAE1* plays a role in the response to drought and heat stresses, whereas *AtSAE2* is essential for plant development [40,41]. Currently, it is not possible to obtain *Atsae1b* null T-DNA insertion plants; therefore, its role remains unknown [31].

**Figure 2:**
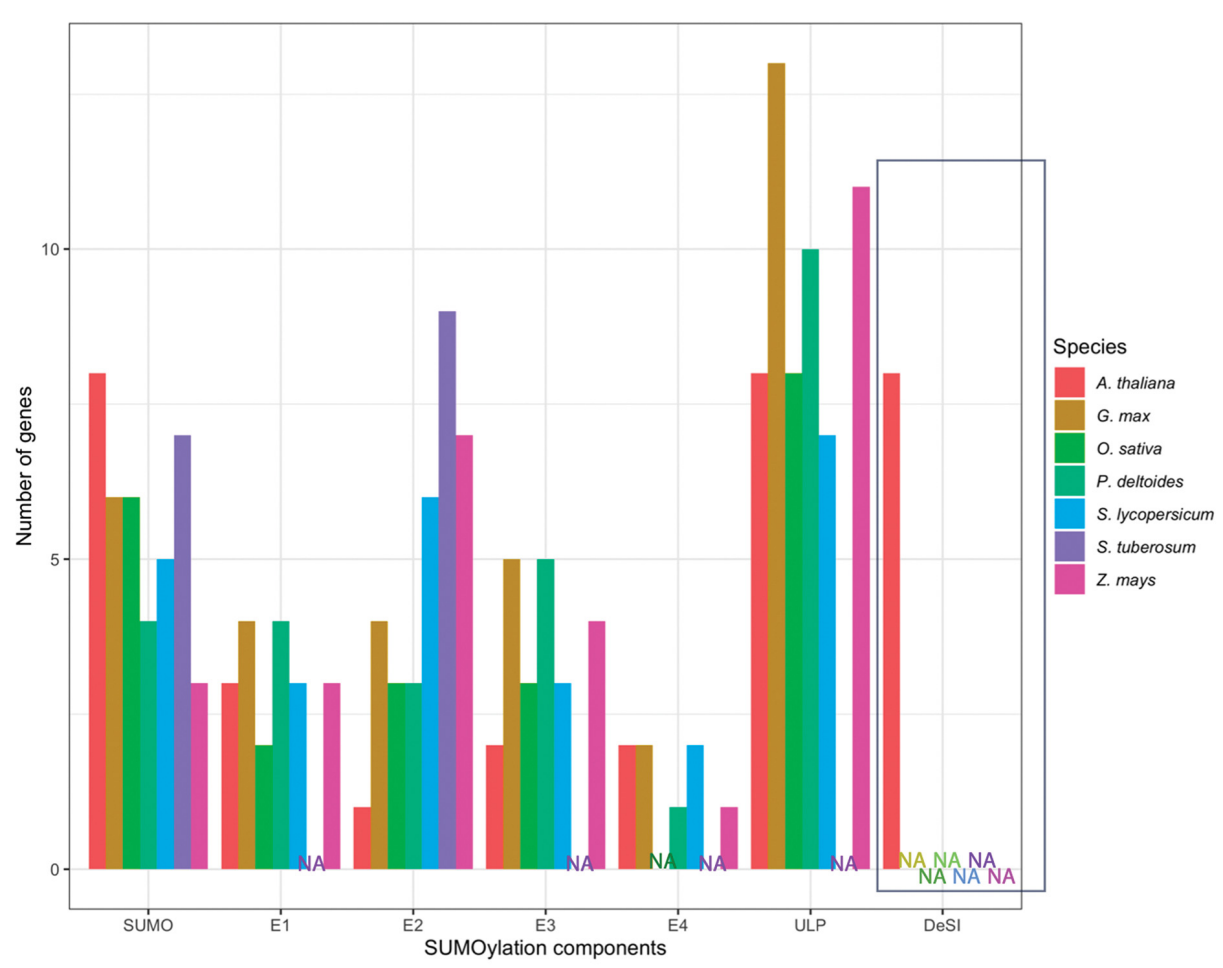
Collective summary of the SUMO components involved in the SUMO cycle of *A. thaliana*, soybean (*G. max)*, rice (*O. sativa*), tomato (*S. lycopersicum)*, potato (*S. turberosum)*, and maize (*Z. mays)* ULP and DeSI genes are SUMO proteases that function to either mature or deSUMOylate SUMOylated proteins; however, only ULP proteases play a role in maturation [2]. E1 and E2 genes are essential in the SUMO cycle as they encode for proteins that are essential to catalyzing the activation of matured SUMO proteins and facilitating their transfer onto a lysine residue in the target protein through a process called SUMO conjugation [29–32]. Although this process can occur directly through E2, E3 ligase genes are also essential as they encode for proteins that further aid in the transfer of the SUMO protein on the target lysine residue—making the process of SUMO conjugation more efficient [25]. Proteins encoded by E4 genes provide a further step in the SUMO cycle that promotes the formation of SUMO chains [33,35]. All data displayed in this figure were gathered from Ghimire et al. [12] and Li et al. [42]. The colors used in this figure each correlate to a separate plant species and display the number of each of the SUMO machinery components that have been found in the specific species so far. NA is used where currently no data is available for this component in the relevant species.

Currently, there are several known genes encoding for E1 enzymes in common crops; however, there is no indication of the presence of E1 genes in *S. tuberosum* but this is likely due to the SUMO machinery not yet being completely described in this species—Figure 2. While no data have currently been reported on the role of E1 in response to stress in the majority of the major crops, some data have been recorded in *G. max*. The results showed that levels of *GmSAE1a* were slightly increased following 6 h of salinity treatment—with levels returning to normal after 24 h. A similar pattern of expression was also observed under heat-shock treatment [42]. Although these results are suggestive that *GmSAE1a* may play a role in stress response, it is unclear as to what phenotypic advantage it offers. This is a common theme among E1 enzymes in crops; therefore, more research is required to understand their role during stress and if the addition of more E1 enzymes constitutes an evolutionary advantage.

**Figure 3:**
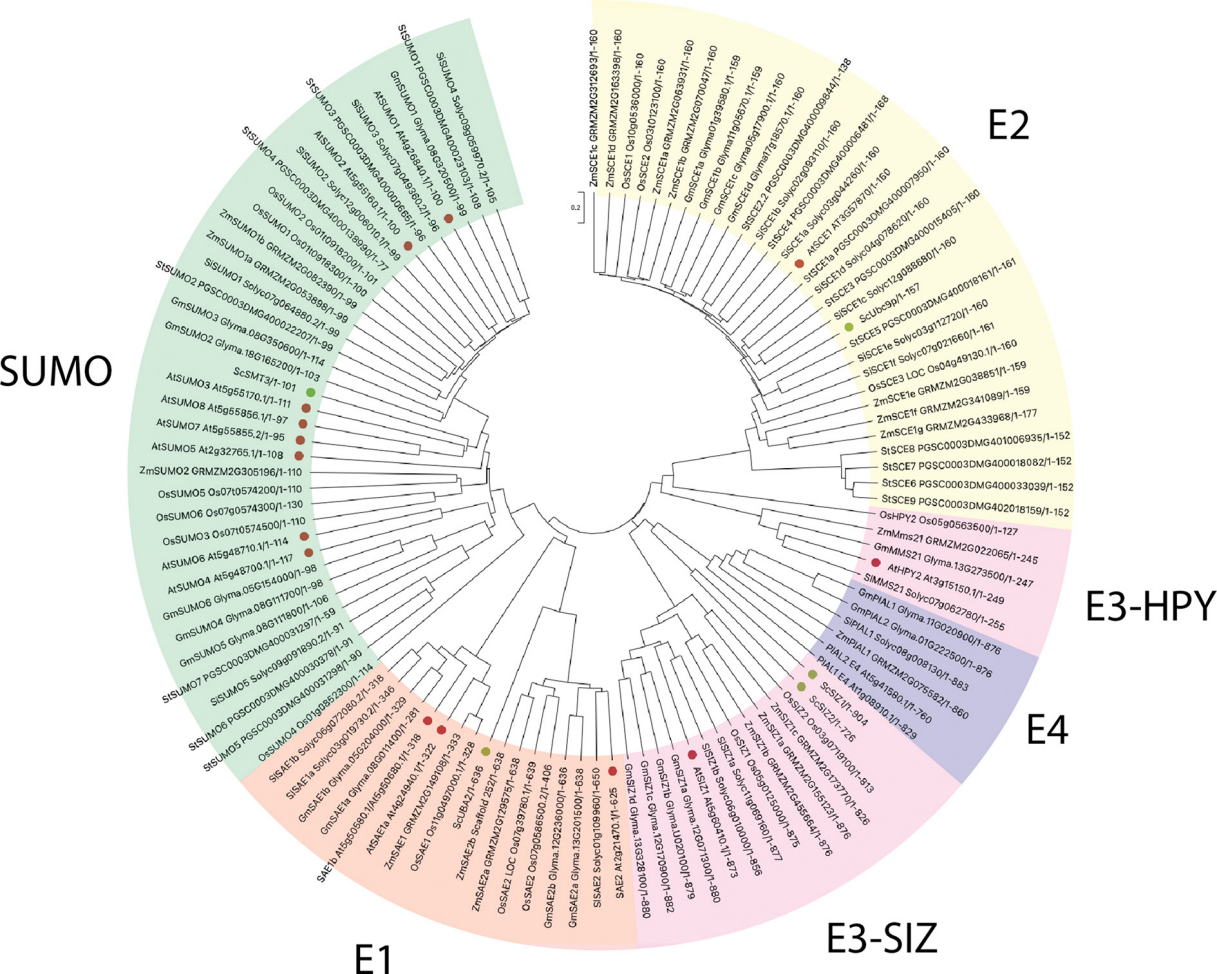
Phylogenetic tree of SUMO, E1–E4 of *A. thaliana*, *G. max*, *O. sativa*, *S. lycopersicum*, *S. turberosum*, and *Z. mays* Protein sequences from Table 1 were aligned by MUSCLE, constructed, and visualized via a phylogenetic tree by MEGA 11, using the Neighbor Joining method with 1000 bootstraps. Red dots and green dots represent protein sequences from *A. thaliana* and yeast, respectively. All displayed data were gathered from Ghimire et al. [12] and Li et al. [42].

While searching the literature, we also found that low copy numbers of SAE genes in *A. thaliana* and the major crops, excluding *S. tuberosum*, can be ascribed to single copies of these genes in common ancestors of dicots and monocots [43]. In addition, E1 enzymes are closely related among the six species, which is demonstrated in Figure 3, where branches are much shorter compared with other SUMO components. Another notable feature is that the E1 subunits observed in *A. thaliana* are identical with those observed in *S. lycopersicum*. It could be speculated that perhaps the E1 subunits in these two species operate in a similar way; however, there is currently no literature demonstrating the role of E1 in *S. lycopersicum*. A final observation to make is that the species *G. max*, similar to *A. thaliana*, also has two SAE1 subunits—*GmSAE1a* and *GmSAE1b*; however, the *GmSAE2* subunit in *G. max*, as opposed to *A. thaliana*, is divided further into two subunits—*GmSAE2a* and *GmSAE2b*—Table 1. As knockouts of *AtSAE2* are embryo lethal, could the division of *GmSAE2* be an evolutionary development to enhance stress survival or could it be that more E1 genes are required in crop species due to their need for another layer of regulation?

## E2-conjugating enzymes

*AtSCE1* is the only SUMO-conjugating E2 enzyme described in *A. thaliana*. This enzyme is critical within the SUMO cycle due to its role in SUMO conjugation and *AtSCE1* mutants being embryonic lethal—arresting early during embryo development [41]. As *AtSCE1* is required during early development, the literature is limited regarding the role of *AtSCE1* during stress; however, multiplication of E2-conjugating enzymes and their role within stress in crops is slightly better established.

Compared with *A. thaliana*, in crops, there are several genes encoding SCE1—Table 1 and Figure 2. This expansion of SCE genes may constitute an evolutionary advantage to produce higher stress and disease-tolerant crops and is likely due to tandem duplication in some crops and the tetraploid nature of *S. tuberosum* [44,45]. This is evident in the literature where overexpression of *OsSCE3* has shown to enhance drought tolerance with a stimulated growth recovery of 90–96% observed after 2 days of drought compared with WT with a recovery rate of 40–64% [46]. *G. max* transcript levels of *GmSCE1a* and *GmSCE1d* have also shown to be up-regulated during stress with *GmSCE1d* transcript levels significantly increased as early as 1 h, following salinity stress and *GmSCE1a* exhibiting a faster response during heat-shock treatment compared with *GmSCE1d*. A role for *GmSCE1a* in immunity was also observed with transcript levels up-regulated, following *Phytophthora sojae* infection [42,47]. The *S. tuberosum* SCES—*StSCE1, StSCE5, StSCE6, and StSCE7* transcript levels all increased during both salinity and PEG-induced water stress; however, *StSCE9* was up-regulated during salinity stress and down-regulated during PEG-induced water stress [48]. *Solannum peruvianum*, a wild species of tomato, also showed that silencing of *SpSCE1* leads to an increase in disease vulnerability to *Clavibacter michiganensis ssp. michiganensis* [49]. Although informative, the majority of E2 data in crops lacks phenotypic observations.

Initial results in *Z. mays* indicated that *ZmSCE1e* transcript levels increased during both salinity and PEG-induced water stress. Using these initial findings, Wang et al. [50] went on to experimentally overexpress *ZmSCE1* in three different transgenic tobacco lines and conferred tolerance to drought. After 15 days in the absence of water, WT tobacco plants demonstrated severe wilting, whereas the *ZmSCE1e* transgenic tobacco plants showed only moderate signs of stress with most of the upper leaves remaining green. After rewatering, nearly 50% of all WT plants died, whereas all transgenic lines started to grow again. More recently, Wang et al. [51] also overexpressed *ZmSCE1d* in four transgenic *A. thaliana* lines and were able to again confer drought tolerance and observed similar results. Following 17 days without water, the majority of WT plants experienced severe wiliting or lethality, whereas transgenic *ZmSCE1d* lines showed only moderate water deficit with 50% of upper leaves remaining green and fully expanded. These initial results suggest that SCEs may have a conserved function in stress protection. We speculate that SCEs may offer a SUMO-dependent mechanism for crop protection under stress. Phylogenetic analysis of the SUMO machinery in both *A. thaliana* and crops reveals the conservation of protein sequences within each SUMO component among the six species—Figure 3.

## E3 ligases

Unlike *Atsae2* and *Atsce1* knockouts, single mutants of the SUMO E3 ligases are viable and in fact, have shown to be effective in several stress responses [53,54]. This is apparent where following exposure to 45°C for 1 h, *AtSIZ1* WT 5-day-old seedlings developed severe chlorotic whereas, under the same conditions, *Atsiz1* mutant seedlings remained healthy [55]. *AtSIZ1* has also been found to negatively regulate immunity in *A. thaliana* where increased resistance to *P. syringae* pv. Tomato DC3000 (Pst DC3000) was observed in *Atsiz1* mutants due to an elevation in the levels of SA and the up-regulation of immune response genes [56].

In *A. thaliana*, the E3 ligase *AtMMS21/AtHPY2* has been shown to negatively regulate drought tolerance. Zhang et al. [56] observed that following 17 days of drought conditions *Atmms21* knockout *A. thaliana* plants showed no drought stress symptoms, whereas WT plants showed weak drought symptoms and *AtMMS21/AtHPY2* overexpressed plants exhibited severe drought symptoms. The present study suggests that like *AtSIZ1*, *AtMMS21/AtHPY2* negatively regulates stress in *A. thaliana*; however, this pattern of negative regulation is somewhat contradictory in *Atmms21* plants as although mutants are viable and exhibit a level of drought resistance, this is accompanied by severe dwarfism, stunted development in root growth and defective meristem [57]. The main reason for this is due to the role of *AtMMS21/AtHPY2* in repressing the endocycle onset in *A. thaliana* meristem with *Atmms21/Athpy2 mutants* displaying defective meristems accompanied by severe dwarfism due to early transitioning from the mitotic cycle to the endocycle [58]. Although knockout of the E3 ligase *AtMMS21/AtHPY2* is considered viable and beneficial in response to drought stress it is accompanied by undesirable severe defects that are not worth the trade-off. This pattern of negative regulation, however, is not observed in *S. lycopersicum*, *Z. mays*, and *G. max* where overexpression of *SiSIZ1* in *S. lycopersicum* has shown to boost tolerance during heat stress by reducing the accumulation of reactive oxygen species (ROS) and up-regulating heat-shock transcription factors (HSFs) [11]. In *Z. mays*, up-regulation of *ZmSIZ1a*, *ZmSIZ1b*, and *ZmSIZ1c* transcript levels were observed in response to drought and salinity stress, and in *G. max* GmSIZ1a and GmSIZ1b expression was stimulated in response to heat and dehydration—promoting *GmSUMO1* conjugation [59,60].

The SUMO E3 ligase SIZ1 has also shown to exhibit conserved function similar to E2. By overexpressing *OsSIZ1* in transgenic *A. thaliana* lines, it is possible to increase thermotolerance and tolerance to both drought and salinity stress—a response previously negatively regulated in *A. thaliana* [61]. Similarly, overexpression of *OsSIZ1* in cotton plants has also been shown to increase drought tolerance, improve growth, and increase fibre yield [62]. These results further support the notion that the conserved function of the SUMO machinery may be utilized to confer stress tolerance universally across different crops and plants. Phylogenetic analysis of the E3 ligases SIZ1 and HPY2, unlike other clusters, is placed into two separate groups signifying unique sequences and possible unique functions with null mutants in *AtSIZ1* in *A. thaliana* plants, showing a severe pleiotropic phenotype [32,63]—Figure 3.

## E4 ligases

E4 SUMO ligases function by promoting SCE1-dependent SUMO chain formation and are currently not as vastly identified in crops in comparison with *A. thaliana* as observed in other SUMO components—Table 1. In *A. thaliana*, Tomanov et al. [35] observed that *Atpial1* and *Atpial2* mutants displayed better growth compared with WT when under both salinity and osmotic stress as well as exhibiting altered sulfur metabolism. The literature is again limited regarding the role of crop E4 ligases in stress response.

## SUMO proteases

SUMO proteases are essential within the SUMO cycle due to their role in both SUMO maturation and deSUMOylation. So far seven DeSI and eight ULP proteases have been identified in *A.thaliana* [24]—Table 1. Two ULPs that have been shown to confer tolerance to several stresses are *AtOTS1* (overly tolerant to salt1) and *AtOTS2*. These ULPs were initially identified in response to salinity stress where it was observed that during high-salt growth conditions, *AtOTS1/2* were degraded to regulate salt stress response, whereas *Atots1 Atots2* double mutants displayed extreme sensitivity to salt exposure [64]. Similarly, *AtOTS1/2* are also responsible for the deSUMOylation of *auxin response factor 7* (*ARF7)* to provide roots with hydropatterning during wet environments by forming lateral roots in the direction of water—regulating osmotic stress. [65]. Aside from abiotic stresses, *AtOTS1/2* have also been shown to play a role in immunity, where Bailey et al. [66] found that the double mutant *Atots1 Atots2* displayed elevated levels of conjugated SUMO, SA, and increased resistance to Pst DC3000 compared with WT plants. In their WT form, the SUMO proteases *AtOTS1/2* limit the production of SA by suppressing the expression of *isochorismate synthase1* (*ICS1*) and as a feedback loop are degraded upon SA treatment to regulate SA signalling.

Similarly to *AtOTS1/2*, the ULP protease *Atesd4-1* mutant is also influenced by the of *ICS1* with overexpression leading to an increase in the accumulation of SA [25,36]. The ULPs *AtSPF1* and *AtSPF2* also play a role in immunity by mediateing the SUMOylation of WRKY33 during flg22 treatment and infection from *Botrytis cinerea*. SUMOylation mediates the phosphorylation of WRKY33 facilitated by the interaction of mitogen-activated protein kinases (MAPKs), MAPK3 and MAPK6 [13].There is limited information known regarding the role of *DeSI* proteases in the model plant; however, the *AtDesi3a* protease has been shown to play a critical role in *FLS2*-mediated immunity. Orosa-Puente et al. [67] found that in *A. thaliana*, after induction of flagellin, *AtDesi3a* was degraded that promoted the SUMOylation of *FLS2* to promote the dissociation and release of BIK1 from the *FLS2* complex and thus activated PTI intracellular immune signalling.

When it comes to the role of SUMO proteases in the response to stress in crops very little is known; however, recent literature has shown an expansion of ULPs in *O. sativa* where a role for the SUMO proteases *OsOTS1* and *OsOTS2* has been observed during drought and salinity stresses. Transgenic-rice plants overexpressing *OsOTS1* have been shown to enhance salt tolerance with an increase in the levels of salinity shown to trigger degradation of *OsOTS1*—indicating that SUMO conjugation in rice plants during salinity stress is due to down-regulation of *OsOTS1/2* activity [68]. *OsOTS1* has also been shown to mediate drought tolerance in *O. sativa* plants. This is evident where knockout of *OsOTS1* in transgenic plants leads to an increase in the accumulation of ABA and more productive agronomic traits during drought stress, whereas *OsOTS1* overexpressing lines displayed increased drought-sensitivity and ABA insensitivity [69]. In current literature, there is no identification of DeSI proteases in any crops due to their recent characterization in *A. thaliana* [2,70].

## The implication for SUMO in future-proofing crops against climate change

There is relatively less information available about the role of SUMO in crops; therefore, in the present review, we provided an outline of the SUMO components in crops and how these provide a mechanism of defence against biotic and abioitic stresses in comparison with the model organism *A. thaliana*. SUMO is an essential PTM that is a multifaceted process that plays an essential role in the regulation of several stress responses in plants and major crops [25]. It is evident that the number of SUMO components, especially E2, E3, and ULP genes, have increased in major crops in comparison with *A. thaliana* [11,42]—Table 1 and Figure 2. This increase is likely ascribed to an evolutionary advantage in crops to enable their survival as it has shown that polyploidy offers benefits to some physiological characteristics by providing higher stress and disease tolerance; however, this could also be due to the requirement of extra SUMO genes in crop species to provide an extra layer of regulation that is not needed in *A. thaliana* [50,51,71]. Phylogenetic analysis of E2 SCE genes in crop species shows that there has been an independent duplication of E2 SCE genes, suggesting that crops have a greater reliance on the SUMO system for yield stability under environmental stress—Figure 3.

Multiple studies have successfully demonstrated the conserved function of SUMO in stress tolerance across different species. As E2 genes are not closely related among the different crops and *A. thaliana* on the phylogenetic tree it suggests that perhaps it is not required that species must be closely related to take advantage of this conserved function. Therefore, core stress response pathways in different species can be revealed by studying their SUMO-dependent mechanisms and this knowledge can readily be transferred to different crops to provide the key tools needed to produce stress-resistant crops to maintain global security.

A final point to make is regarding the limited information available on the role of SUMO proteases in crops. In *A. thaliana*, the previous literature has shown that SUMO proteases play a role in multiple abiotic and biotic stresses; however, the protease enzymes in crops are not well-characterized and their function remains unknown aside from the role of some ULPs in rice and maize [70–72]. Currently, it is known that there are 12–22 ULP proteases in rice; however, only seven *OsULPs* have been tested for protease function [69,71–74]. It has been speculated that the expansion of ULPs in crops could have resulted from domestication and could give rise to target specificity during deSUMOylation [11,72,73,75]. As for DeSI proteases, to our knowledge, there is currently none identified in crops due to the recent characterization in *A. thaliana*; however, with more importance being placed on SUMO and its potential use in future-proofing crops, this is likely to change [2,70].

## Summary

- SUMO plays a critical role in stress responses in the model plant *A. thaliana* and major crops.
- Major crops have an increase in SUMO genes compared with *A. thaliana*.
- The SUMO reveals conserved core stress response pathways in model plants and crops.
- Identifying SUMOylated targets regulating these core responses could pave the way to generating stress-tolerant crops.

## Open Access

Open access for the present article was enabled by the participation of Durham University in an all-inclusive *Read & Publish* agreement with Portland Press and the Biochemical Society under a transformative agreement with JISC.

## Abbreviations

ARF7: auxin response factor 7
FLS2: flagellin-sensitive 2
BIK1: botrytis-induced kinase1
Pst DC3000: Pseudomonas syringae pv. tomato DC3000
WT: wild-type
A. thaliana: Arabidopsis thaliana
At: A. thaliana
Gm: G. max
Os: O. sativa
Si: S. lycoperisium
St: S. tuberosum
Zm: Z. mays
HPY2: high ploidy2
HSF: heat-shock transcription factor
ICS1: isochorismate synthase1
MAPK: mitogen-activated protein kinase
NPR1: nonexpressor of pathogenesis related 1
OTS1: overly tolerant to salt1
SIZ1: sap and miz1
PIAL1: protein inhibitor of activated state like1
PTM: post-translational modification
ROS: reactive oxygen species
SA: salicylic acid
SAE: SUMO-activating enzyme
SCE: SUMO-conjugating enzyme
SMT3: suppressor of mif two3
SUMO: small ubiquitin-like modifier
UPS: ubiquitin-proteasome system
